# Auburn Notes

**Published:** 1915-11

**Authors:** 


### AUBURN NOTES

Dr. L. Belle Richens is recovering from an attack of typhoid fever.

---

Dr. R. W. H. Rollings has opened an office at 36 Fulton Street.

---

Dr. and Mrs. S. W. Day accompanied by Dr. and Mrs. Geo. C. Sincerbeaux motored to New York City for a weeks stay.

---

Dr. Thomas F. Laurie is spending a short time in New York City in postgraduate study.

---

The recent typhoid epidemic, milk-borne infection, is practically at an end.

---

Dr. Frederick Sefton was the principal speaker at the latest meeting of the Auburn City Medical Society, and related his recent experiences in England and France.

---

**Saccharomyces Simulating Pulmonary Tuberculosis.** Percy K. Telford, of Los Angeles, ibid. sends culture and refers to 7 cases reported by Lotena M. Breed in the J. A. M. A., 3 years ago. Vaccine and KI are advocated as treatment.

